# Translational regulation by ribosome-associated quality control in neurodegenerative disease, cancer, and viral infection

**DOI:** 10.3389/fcell.2022.970654

**Published:** 2022-09-14

**Authors:** Bingwei Lu

**Affiliations:** Department of Pathology, Stanford University School of Medicine, Stanford, CA, United States

**Keywords:** ribosome-associated quality control, neurodegenerative diseases, cancer, viral infection, COVID-19, proteostasis, translation stalling, ribosome collision

## Abstract

Translational control at the initiation, elongation, and termination steps exerts immediate effects on the rate as well as the spatiotemporal dynamics of new protein synthesis, shaping the composition of the proteome. Translational control is particularly important for cells under stress as during viral infection or in disease conditions such as cancer and neurodegenerative diseases. Much has been learned about the control mechanisms acting at the translational initiation step under normal or pathological conditions. However, problems during the elongation or termination steps of translation can lead to ribosome stalling and ribosome collision, which will trigger ribosome-associated quality control (RQC) mechanism. Inadequate RQC may lead to the accumulation of faulty translation products that perturb protein homeostasis (proteostasis). Proteostasis signifies a cellular state in which the synthesis, folding, and degradation of proteins are maintained at a homeostatic state such that an intact proteome is preserved. Cellular capacity to preserve proteostasis declines with age, which is thought to contribute to age-related diseases. Proteostasis failure manifested as formation of aberrant protein aggregates, epitomized by the amyloid plaques in Alzheimer’s disease (AD), is a defining feature of neurodegenerative diseases. The root cause of the proteostasis failure and protein aggregation is still enigmatic. Here I will review recent studies supporting that faulty translation products resulting from inadequate RQC of translational stalling and ribosome collision during the translation of problematic mRNAs can be the root cause of proteostasis failure and may represent novel therapeutic targets for neurodegenerative diseases. I will also review evidence that translation regulation by RQC is operative in cancer cells and during viral infection. Better understanding of RQC mechanism may lead to novel therapeutic strategies against neurodegenerative diseases, cancer, and viral infections, including the ongoing COVID-19 pandemic.

## Introduction

Translation is the cellular process that converts the genetic information encoded in mRNAs to the thousands of proteins essential for life by a large macromolecular machine called ribosome. The ribosome reads the information one codon at a time, through the action of tRNAs that recognize each codon and insert the specified amino acids into proteins. This process is conserved in all domains of life (Dever and Green, 2012; Hershey et al., 2019). Translation is an energy-demanding process. Up to 20% of cellular energy is dedicated to translation in normal proliferating cells as opposed to 15% for DNA replication and transcription combined (Buttgereit and Brand, 1995). Translational control therefore responds to cellular metabolic state and is critical for cellular stress response, and misregulation of this process often leads to disease (Tahmasebi et al., 2018).

Translation can be divided into four main stages: initiation, elongation, termination and ribosome recycling (Dever and Green, 2012). Although core aspects of translation are highly conserved between eukaryotes and prokaryotes, there are differences at the detailed biochemical level in how each of the four steps is accomplished. In the translation initiation step, eukaryotic translation initiation factors (eIFs) guide the assembly of an 80S ribosome at the AUG start codon with an initiator methionyl-tRNA bound. This is achieved by formation of the eIF4F complex composed of eIF4A, eIF4E, and eIF4G at the 5′ end of mRNAs that acts to unwind mRNA tertiary structures, the tertiary complex (TC) composed of eIF2-GTP-Met-tRNA^Met^, and the formation of the 43S preinitiation complex consisting of 40S ribosome, TC, and other eIFs such as eIF3 and eIF1. The 43S preinitiation complex then scans the 5′-UTR sequence in the 5′ to 3′ direction to find the initiation codon. After initiation codon recognition, the 60S large ribosome is joined and 80S ribosome is formed (Sonenberg and Hinnebusch, 2009). During elongation, 80S ribosomes move vectorially along the mRNA, one codon at a step, synthesizing the encoded protein one amino acid at a time through the coordinated actions of aminoacyl-tRNAs, eukaryotic elongation factors (eEFs), and peptide bond formation at the peptidyl transferase center (Dever et al., 2018). The tRNAs vectorially transition between three sites inside the ribosome: the A site, P site and E site. At the end of the open reading frame, the ribosome encounters a termination codon encoded by UAG, UAA, or UGA, which is specifically recognized by a set of protein factors called eukaryotic peptide chain release factors (eRFs) including eRF1 and eRF3, which promote the release of the nascent peptide chains from the peptidyl-tRNA (Hellen, 2018). In the final recycling phase, the post-termination 80S ribosome complex is dissociated with the energy released from ATP hydrolysis by the ATP-binding cassette subfamily E member 1 (ABCE1) protein into 40 and 60S subunits to begin a new round of translation (Jackson et al., 2012; Mancera-Martinez et al., 2017).

### Ribosome stalling, collision, and ribosome-mediated quality control

**FIGURE 1:**
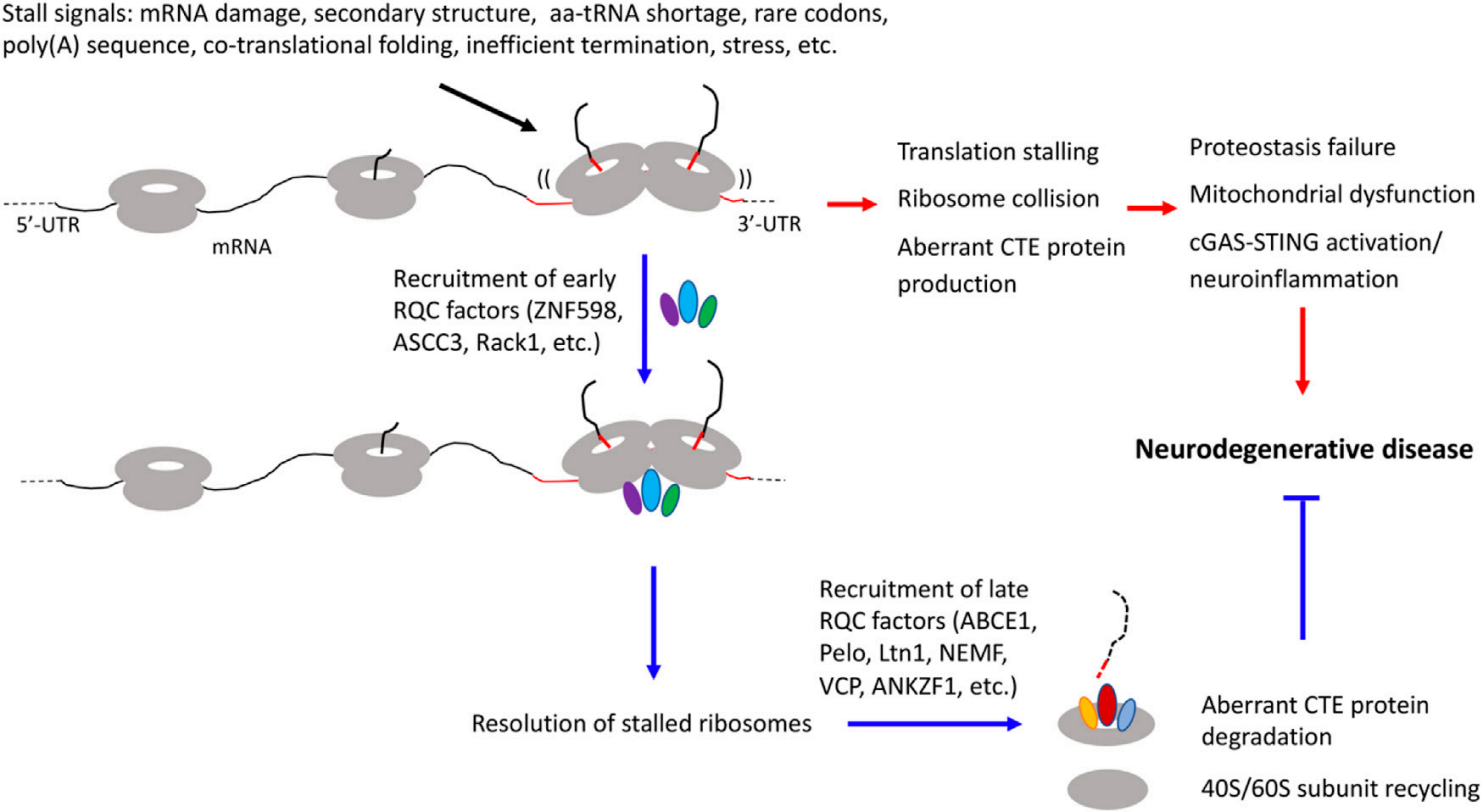
Diagram depicting diverse signals that can lead to translation stalling and ribosome collision and the effect of RQC factor recruitment to stalled ribosomes to resolve stalled translation and restoring cellular homeostasis, and how inadequate quality control of stalled translation and collided ribosomes may be linked to various hallmarks of neurodegenerative diseases, including proteostasis failure, mitochondrial dysfunction, and neuroinflammation.

During translation, ribosomes do not proceed at a constant speed. Ribosome slowdown and stalling can occur for various reasons. Some of these events are functional and serve to facilitate cellular dynamics, such as co-translational protein folding, subcellular protein targeting, and mRNA localization (Stein and Frydman, 2019). Others are detrimental and can be triggered by translation of mRNAs with aberrant features such as long polyA sequences resulting from mRNAs without a stop codon or with altered polyadenylation, mRNA damages, and higher order mRNA structures. Insufficient supply of aminoacyl-tRNAs, inefficient ribosome termination/recycling, or environmental stresses such as UV irradiation can also lead to ribosome stalling/collision (Ito-Harashima et al., 2007; Bengtson and Joazeiro, 2010; Harigaya and Parker, 2010; Buskirk and Green, 2017; Wu et al., 2019; Wu et al., 2020). Ribosome slowdown and stalling can result in ribosome collision, which is sensed by the cell as a proxy for aberrant translation and triggers ribosome-associated quality control (RQC) (Kim and Zaher, 2022). Key factors involved are the ubiquitin ligase ZNF598 and the 40S subunit protein Rack1, which recognize the distinct 40-40S interface characterizing collided ribosomes and promote ubiquitination of specific 40S subunit proteins include Rps10, Rps20, Rps2, and Rps3 (Juszkiewicz and Hegde, 2017; Sundaramoorthy et al., 2017), and the ASC-1 complex containing ASCC3, ASCC1, and ASCC2 in mammals and a similar complex in yeast that disassembles the leading collided ribosome (Hashimoto et al., 2020; Juszkiewicz et al., 2020). This then triggers a series of downstream quality control events, including ribosome subunit splitting and recycling by ABCE1 (Shao and Hegde, 2014), modification of the nascent peptide chains (NPCs) still attached to the 60S subunit by a C-terminal Ala and Thr addition (CAT-tailing) process (Shen et al., 2015), release of nascent peptide chain from the 60S complex by ANKZF1/VMS1 (Verma et al., 2018), and degradation of the aberrant translation products by the Ltn1/VCP/NEMF complex (Brandman et al., 2012) (Figure 1). Although the exact physiological role of the CAT-tails remains to be established, with functions in serving as a degron to promote aberrant NPC degradation (Lytvynenko et al., 2019; Sitron and Brandman, 2019), or pushing K residues residing in the ribosome exit channel for ubiquitination by Ltn1 (Kostova et al., 2017) having been proposed, their accumulation under impaired RQC conditions can perturb proteostasis due to their intrinsic ability to form detergent-insoluble protein aggregates (Choe et al., 2016; Yonashiro et al., 2016). Moreover, recent studies link ribosome stalling/collision with integrated stress response (Ishimura et al., 2016; Harding et al., 2019; Inglis et al., 2019; Meydan and Guydosh, 2020; Yan and Zaher, 2021), suggesting that deregulated integrated stress response may also contribute to the cellular toxicity induced by unresolved ribosome stalling/collision.

## Pathogenic role of inadequate ribosome-associated quality control in neurodegenerative diseases

Studies of protein aggregation have historically been focused on alterations of post-synthesis, mature proteins. Such changes emphasize posttranslational modifications (phosphorylation, ubiquitination, acetylation, oxidation, cleavage, etc.), and significant efforts have been made to link these molecular events to aging-related cellular dysfunctions such as oxidative stress (Butterfield, 2018) and impairments of the autophagy and ubiquitin-proteosome systems (Hohn et al., 2020). Recent studies provide compelling evidence that problems of proteostasis can begin with nascent peptide chains (NPCs) still associated with translating ribosomes, necessitating the deployment of RQC to handle translation problems and prevent the accumulation of faulty translation products (Brandman and Hegde, 2016; Joazeiro, 2019).

### Ribosome-associated quality control in Parkinson’s disease

Previous studies of ribosome stalling, RQC, and CAT-tailing have made extensive use of artificial substrates such as mRNAs with no stop codons or mRNAs containing rare codons or encoding long-stretches of basic amino acids, and such studies are largely done in single cell systems including yeast and mammalian cell culture (Lykke-Andersen and Bennett, 2014). Studies in the *Drosophila PINK1* model of Parkinson’s disease (PD) showed, for the first time in metazoans, that aberrant RQC can lead to CAT-tailing like modification of mitochondrial proteins, leading to impairment of proteostasis and contributing to neurodegeneration (Wu et al., 2019). Parkinson’s disease is a neurodegenerative disease and the most common movement disorder characterized by loss of dopaminergic neurons in the substantia nigra region of the mid-brain, although neurons in other brain regions are also affected. Familial mutations in Pten-induced kinase 1 (PINK1) and Parkin are associated with early onset PD (Kitada et al., 1998; Valente et al., 2004). PINK1 encoded a mitochondrial Ser/Thr kinase that works together with Parkin, an E3-ubiquitin ligase, to regulate mitochondrial function and mitochondrial quality control by mitophagy (Narendra et al., 2008; Narendra et al., 2010; Youle and Narendra, 2011), with PINK1 acting upstream of Parkin as first demonstrated in flies (Clark et al., 2006; Park et al., 2006; Yang et al., 2006). One of the functions of the PINK1/Parkin pathway is to promote the co-translational import of complex-I 30 kD subunit protein (C-I30, also known as NDUFS3) (Gehrke et al., 2015). It was found that C-I30 protein abundance was reduced in *PINK1* or *Parkin* mutant flies or when wild-type animals were exposed to mitochondrial toxins such as rotenone and CCCP. Detailed analysis of C-I30 protein revealed the generation of an upshifted C-I30 band (C-I30-u) under these conditions (Wu et al., 2019). After extensive analyses excluding posttranslational modifications (ubiquitination, phosphorylation, mitochondrial targeting sequence-cleavage) and alternative splicing as possible sources of C-I30-u generation, and after demonstration of C-terminal extension (CTE) as possible mechanism of C-I30-u formation, it was discovered that C-I30 was modified by a CAT-tailing like phenomenon termed mitochondrial-stress-induced translational termination impairment and protein carboxyl terminal extension (MISTERMINATE) (Wu et al., 2019). Moreover, it was found that some CAT-tailed C-I30 proteins were imported into mitochondria, became assembled into respiratory chain complex-I, and caused mitochondrial energy deficit. Other CAT-tailed C-I30 proteins were released into the cytosol where they formed protein aggregates and disrupted proteostasis. Interestingly, distinct from the CAT-tails in yeast that consist exclusively of Ala and Thr (Shen et al., 2015), the CTEs of C-I30 in *PINK1* mutant also contain other amino acids such as Ser, Tyr, Cys, and Glu/Pro as determined by mass spec analysis (Wu et al., 2019). A later study in mammalian cells confirmed the diversity in amino acid composition of CAT-tails (Udagawa et al., 2021). The CTE in C-I30-u is added to C-I30 protein by ribosomes stalled at the canonical stop codon site. Mechanistically, this stalling was shown to be caused by mitochondrial stress-induced impairment of translation termination factor eRF1 and the ribosome recycling factor ABCE1 (Wu et al., 2019). Thus, although the CTE in C-I30-u in metazoans is generated by a mechanism thematically related to CAT-tailing in yeast, there are fundamental differences. Further studies showed that the CTE of C-I30 under mitochondrial stress was observed both *in vivo* in flies and in mammalian cell culture. Importantly, genetic manipulations boosting the activities of ABCE1/eRF1 and other RQC factors, or inhibiting factors involved in CAT-tailing, including NEMF or Ala and Thr tRNA synthetases effectively rescued the neuromuscular degeneration in *PINK1* flies (Wu et al., 2019), supporting the contribution of aberrant RQC of stalled translation and CAT-tailing to PINK1 pathogenesis. It is possible that this RQC-related function of PINK1 and its well-established role in mitophagy likely reflect different aspects of PINK1 function in the continuum of mitochondrial homeostasis maintenance by PINK1.

### Ribosome-associated quality control in C9-ALS/FTD

ALS, or amyotrophic lateral sclerosis, is a progressive neurodegenerative disease characterized by loss of motor neurons in the brain and spinal cord. Many genes have been linked to the pathogenesis of ALS (Taylor et al., 2016). Expansion of GGGGCC (G4C2) hexanucleotide repeats in *chromosome 9 open reading frame 72 (C9ORF72)* gene is the most common genetic cause of ALS with frontotemporal dementia (C9-ALS/FTD), with repeat number ranging from a few dozen to thousands (DeJesus-Hernandez et al., 2011). Possible mechanisms of disease pathogenesis by G4C2 repeat expansion include haplo-insufficiency of C9ORF72, toxicity associated with RNA foci formed by sense and anti-sense RNAs, or proteotoxicity induced by dipeptide repeat (DPR) proteins translated from G4C2 repeat-carrying transcripts (Gendron and Petrucelli, 2018). There is increasing evidence that emphasizes the contribution of DPR toxicity in C9-ALS/FTD, especially arginine-containing DPR proteins (GR and PR) (Freibaum and Taylor, 2017), and possible mechanisms involving diverse cellular processes have been proposed (Gao et al., 2017). In studies of mechanism of poly(GR) pathogenesis, it was discovered that CAT-tailing-like modification of poly(GR) contributes to disease (Li et al., 2020). It was found that poly(GR) could mimic a mitochondrial-targeting signal, causing some poly(GR) to be co-translationally imported into mitochondria. However, poly(GR) translation on mitochondrial surface is frequently stalled, presumably caused by electrostatic interaction between the positively-charged R residues with the negatively-charged residues lining the ribosome exit tunnel. This triggered RQC and CAT-tailing-like CTE, the composition of which was verified by mass spec. CAT-tailing-like CTE was shown to promote poly(GR) stabilization, aggregation, and toxicity. To further dissect the regulatory mechanism involved in the RQC of poly(GR) translation, genetic modifier screening in *Drosophila* was employed. Such genetic studies uncovered a mitochondria-associated non-canonical Notch signaling pathway that impinges on the RQC machinery to restrain poly(GR) accumulation, in part through AKT-mediated phosphorylation of RQC component VCP. The non-canonical Notch signaling pathway and other genetic modifiers uncovered from the *Drosophila* studies were found to perform similar roles in C9ALS/FTD patient cells (Li et al., 2020), supporting their fundamental involvement in and relevance to ALS/FTD.

### Ribosome-associated quality control in Alzheimer’s disease

Alzheimer’s disease (AD) is a looming public health crisis. This progressive, neurodegenerative disorder and the most common form of dementia is affecting more than 40 million people worldwide, with the number projected to double by 2025 if no effective treatment becomes available by then. While a small portion of AD cases is caused by familial genetic mutations, the majority is sporadic with no known cause. At the neuropathological level, AD is characterized by the presence of extracellular plaques composed of beta-amyloid (Aβ) and neurofibrillary tangles composed of hyperphosphorylated tau (Long and Holtzman, 2019). Aβ is derived from the amyloid precursor protein (APP) through a multifaceted proteolytic process (Nhan et al., 2015). Human genetics studies supported that aberrant APP synthesis or processing is central to AD pathogenesis (Karch et al., 2014), but the key etiological driver of disease remains elusive. Despite tremendous amount of research and pharmaceutical efforts that have been focused on Aβ, the inconclusive results of clinical trials targeting Aβ peptides suggest that the key pathogenic species remain to be identified.

Previous studies in mammalian cell culture, mouse transgenic models, and human induced pluripotent stem cell (iPSC)-derived neuron culture suggested that APP C-terminal fragment (APP.C99) can cause toxicity in an Aβ-independent manner (Jiang et al., 2010; Lauritzen et al., 2012; Hung and Livesey, 2018; Jiang et al., 2019; Kwart et al., 2019). The mechanism of APP. C99 pathogenesis is incompletely understood. In studies using *Drosophila* models expressing 1) APP. C99 with the native ER-targeting signal of human APP, 2) full-length human APP only, or 3) co-expressing full-length human APP and β-secretase (BACE), mammalian cell culture models, mouse 5xFAD model, and postmortem AD patient brain materials to investigate mechanisms of APP. C99 pathogenesis (Rimal et al., 2021), it was found that ribosomes stall at the ER membrane during co-translational translocation of APP. C99. The stall sites were mapped to the stop codon site, and an internal site between the transmembrane domain and the stop codon site. Ribosome stalling at the stop codon site was shown to be due to decreased levels of ribosome recycling and termination factors ABCE1 and eRF1/eRF3, whereas the internal stalling was caused by a combination of factors including ER-targeting, translocon-gating, and subsequent protein folding and membrane insertion (Rimal et al., 2021). These events are intrinsic to APP. C99 biogenesis and membrane topogenesis and likely cause slowdown of translation and ribosome collision. The RQC pathway was activated in cells perhaps in attempt to resolve ribosome collision and stalled translation. Importantly, stalled APP. C99 species resulting from inadequate RQC were found to be prone to aggregation, causing endolysosomal and autophagy defects and seeding the aggregation of Aβ peptides, the main component of amyloid plaques. Genetic manipulations that remove stalled and CAT-tailed APP. C99, such as ABCE1 overexpression or knockdown of ZNF598, rescued proteostasis failure, endolysosomal/autophagy dysfunction, neuromuscular degeneration, and cognitive deficits in the AD models (Rimal et al., 2021). Importantly, many RQC factors such as NEMF, ANKZF1, ZNF598, and Rack1, but not ABCE1 or eRF1, were found deposited at the core of amyloid plaques from AD patient brains (Rimal et al., 2021), a finding that further supports the central role of defective RQC of ribosome collision and stalled translation in AD pathogenesis. These results raise the interesting possibility that each amyloid plaque may contain at its core a neuron dying of proteostasis failure due to inadequate RQC of stalled APP. C99 translation. Collectively, these findings demonstrate that amyloid plaque formation is the consequence and manifestation of a deeper level proteostasis failure caused by inadequate RQC of translational stalling and the resultant aberrantly modified APP. C99 species, previously unrecognized etiological drivers of AD. It is worth mentioning that ribosome stalling is clearly not the only way by which abnormal proteins can be generated in neurodegenerative diseases. For example, it was shown that the RQC pathway had little effect on tau protein level and toxicity (Li et al., 2020), suggesting that toxic tau species associated with AD and other tauopathies are likely generated and causing cellular toxicity by different mechanisms.

### Therapeutic implications for neurodegenerative diseases

A common theme emerging from the above studies is that certain mRNAs encoding disease-associated proteins are physiological substrate of the RQC pathway, and that aberrant protein species containing CAT-like CTEs resulting from defective RQC of stalled translation perturb proteostasis and contribute to cognitive dysfunction and neuromuscular degeneration in animal models. Remarkably, RQC appears to play a particularly important role in maintaining mitochondrial protein homeostasis and mitochondrial function, as indicated by studies in yeast (Izawa et al., 2017) and *Drosophila* (Wu et al., 2019). Thus, defective RQC offers a mechanistic link between proteostasis failure and mitochondrial dysfunction (Lu and Guo, 2020), two pathological hallmarks of neurodegenerative diseases (Figure 1). It is apparent that we are only scratching the surface in terms of understanding the biological consequence of translational stalling and ribosome collision on cellular homeostasis, as recent studies suggest that collided ribosomes may present novel cell signaling platforms on which innate immune response and stress signaling pathways influencing cell survival or cell death decisions are activated (Wu et al., 2020; Wan et al., 2021). Future studies in human iPSC-derived models that express disease-causing proteins at physiological levels, thus obviate drawbacks of non-physiological transgene overexpression commonly associated with animal models, will address questions such as the molecular mechanisms underlying RQC failure in different diseases; the composition of the CTEs added to different stalled translation products; the mechanisms of neurotoxicity induced by collided ribosomes and proteins containing aberrant CTEs, especially their link to stress response pathways such as the integrated stress response; and how is the RQC process linked to environmental stresses and the aging process, which underlie the pathogenesis of sporadic neurodegenerative disease (Frisoni et al., 2021).

The above studies also suggested novel therapeutic strategies for neurodegenerative diseases. For example, in the case of AD, modulating ribosome-mediated QC to get rid of aberrant APP. C99 RQC products, or restoring endolysosomal or autophagy function impaired by aberrant APP. C99 RQC products, deserves serious consideration. Moreover, boosting ER and mitochondrial function, which are expected to indirectly reduce the ribosome stalling of ER translocon-engaged APP. C99, may also prove to be beneficial. A connection between RQC and neurodegenerative diseases has been implicated by genetic studies in mice, where an N-ethyl-N-nitrosourea (ENU)-induced mutation in Listerin caused early onset and progressive neurological and motor deficits and neurodegeneration (Chu et al., 2009); loss of GTPBP2, a binding partner of Pelo, together with mutation in a tRNA specifically expressed in the central nervous system, caused ribosome stalling and widespread neurodegeneration (Ishimura et al., 2014); and mutations in NEMF caused progressive motor neuron degeneration in mice and mutations in NEMF were found in patients with juvenile neuromuscular disease (Martin et al., 2020). However, the critical RQC substrates mediating the neurodegenerative effects associated with the above RQC factor mutations remain to be identified. Therefore, future research efforts addressing this and other fundamental questions have the potential to lead to paradigm shift in our understanding of neurodegenerative disease mechanisms and ultimately to the development of therapeutic strategies applicable to a broad spectrum of diseases.

## Translational regulation and ribosome-associated quality control in cancer

The rapid and continuous proliferation of cancer cells require increased protein synthesis and ribosome content. Deregulated translation initiation in cancer has been well studied (Robichaud et al., 2019). For example, upregulation of the initiation complex eIF4F is observed in cancer, and overexpression of eIF4E is sufficient to cause transformation of fibroblasts (Lazaris-Karatzas et al., 1990). Inactivation of 4EBP, an inhibitor of eIF4E, by mTORC1-mediated phosphorylation is also a common event in cancer (Mamane et al., 2006). Moreover, phosphorylation of eIF2a, an event that negatively regulate tertiary complex formation, was deregulated in cancer, although the exact function of eIF2a phosphorylation in cancer biology is controversial and may be context-dependent (Robichaud et al., 2019).

**FIGURE 2:**
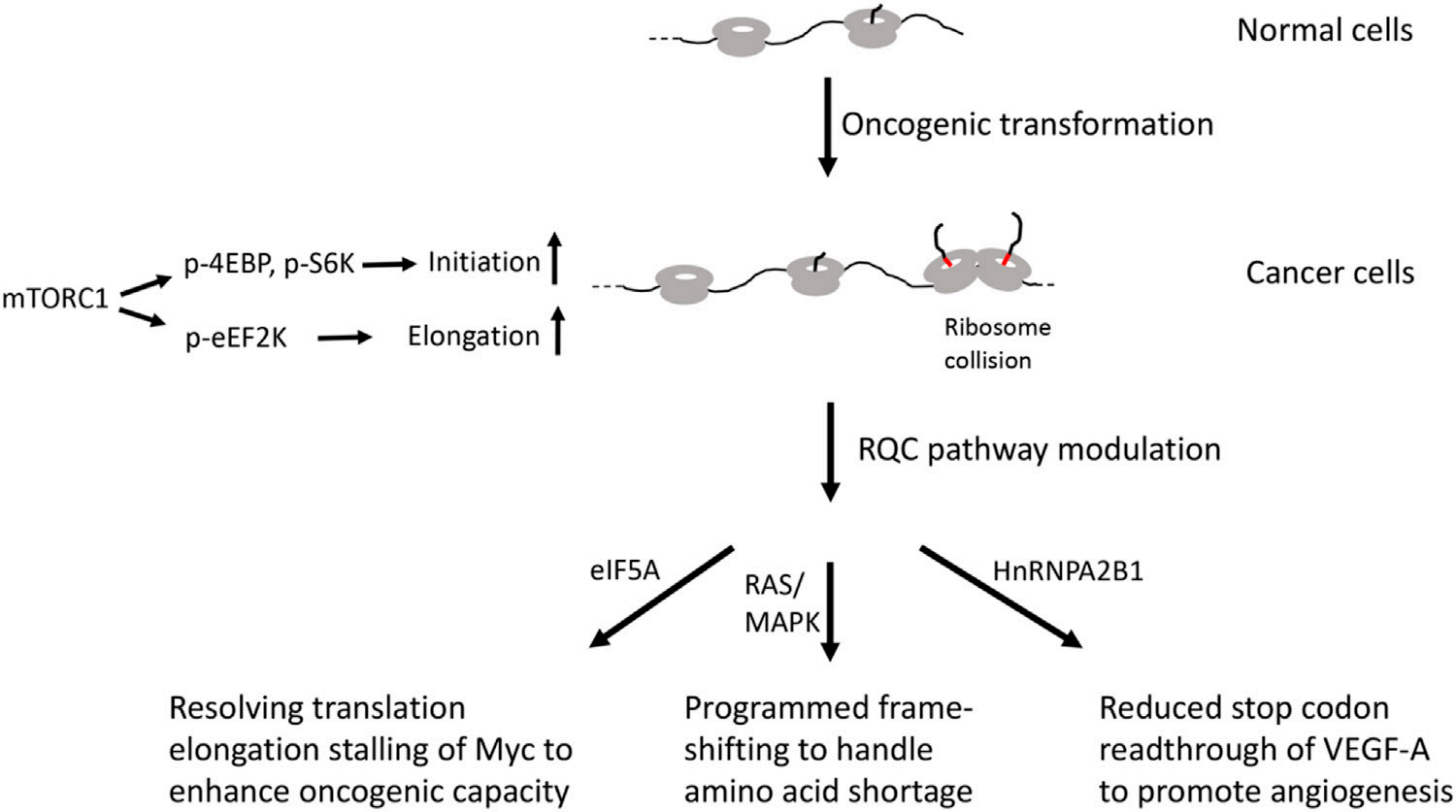
Diagram depicting possible involvement of RQC factors in cancer.

Translational regulation at the elongation and termination steps is also altered in cancer cells. Negative regulation of translation elongation at the eEF2 step by eEF2 kinase (eEF2K) is countered by mTORC1-mediated phosphorylation of eEF2K in cancer cells (Liu and Proud, 2016) (Figure 2). *Cancer* cells overexpress eIF5A, a protein initially identified as an initiation factor but later shown to be important for ribosomes to readthrough difficult-to-translate regions enriched in Pro, Gly, and basic residues (Pelechano and Alepuz, 2017), suggesting that cancer cells may upregulate eIF5A to resolve stalled translation. This is supported by studies in yeast showing that deletion of eIF5A leads to accumulation of stalled ribosomes (Schuller et al., 2017). Interestingly, the transcription factor cMyc, which is a major driver of many types of cancer, contains multiple pausing motifs that affect its biosynthesis (Coni et al., 2020). EIF5A was shown to be involved in the translational elongation of Myc by alleviating ribosome stalls at 5 pausing motifs in the coding sequence of Myc (Figure 2). Inhibition of hypusination, a unique posttranslational modification that regulates the eIF5A activity in alleviating ribosome pausing at specific stall motifs, inhibited the expression of transcript regulated by Myc and inhibited tumor growth in preclinical models of colorectal cancer (Coni et al., 2020). *Cancer* cells also overexpress eIF6 (Brina et al., 2015), an anti-association factor that binds to 60S ribosome at the 40-60S interface and interfere with 40-60S ribosome subunit joining. The exact role of eIF6 in cancer cells remains to be determined. It would be interesting to test if it participates in the handling of stalled or collided ribosomes during the RQC of the peptidyl-tRNA-60S complex by preventing the rejoining of 40S subunit.

### In-frame stop codon readthrough and frame-shifting in cancer cells

Blood vessel development or angiogenesis is critical for cancer growth and survival and represents a promising therapeutic target for cancer. Vascular endothelial growth factor (VEGF)-A is a critical factor that regulates angiogenesis both in physiological and pathologic conditions, including tumorigenesis (Apte et al., 2019). The precise gene dosage of VEGF-A is essential during development, as a 50% reduction or a two-fold increase of VEGF-A dosage can both results in embryonic lethality. Intriguingly, *VEGF-A* mRNA in mammalian endothelial cells undergo programmed stop codon readthrough to generate VEGF-Ax, an isoform containing a 22 amino acid C-terminal extension (Eswarappa et al., 2014). Readthrough is promoted by cis-elements located in the UTR sequence 3’ of the canonical stop codon and the trans-acting RNA-binding protein heterogeneous nuclear ribonucleoprotein (hnRNP) A2/B1 (Figure 2). Importantly, VEGF-Ax possesses anti-angiogenic activity opposite that of VEGF-A, and its expression is depleted in colon adenocarcinoma, suggesting that this translational readthrough event is manipulated by cancer cells to their advantage. Consistent with this notion, genomic-wide studies have revealed higher levels of stop codon readthrough in cancer cells (Wang and Wang, 2021). Although the detailed biochemical mechanism of stop codon readthrough remains to be deciphered, the fact that alteration of RQC activity can result in stop codon readthrough (Annibaldis et al., 2020), and that loss of a recently identified RQC factor EDF1 in yeast can result in frame-shifting at stall sites (Wang et al., 2018), suggests the potential involvement of RQC factors in this process.

In addition to the example of in-frame stop codon readthrough, cancer cells also undergo frame-shifting under amino acid shortage conditions (Figure 2). It was discovered that tryptophan depletion induced ribosomal frame-shifting, allowing ribosomes to bypass the tryptophan codons (W bumps) and continue translation, generating aberrant protein products that are presented at cell surface and allowing recognition by T-lymphocytes, thus exposing a vulnerability of cancer cells that can be exploited therapeutically (Champagne et al., 2021). This ribosomal frame-shifting is associated with oncogenic RAS/MAPK pathway and mediated by the S6K/p-S6 axis, although RAS/MAPK pathway is not sufficient for its occurrence. It remains to be determined whether shortage of other amino acids will induce similar frame-shifting events in cancer cells. Interestingly, studies in yeast have shown that oxidative stress can induce ribosome stalling at tryptophan codons, suggesting that Trp-tRNA availability may be a common cause of ribosome stalling under stress (Rubio et al., 2021). Given the intimate connection between ribosome stalling and frame-shifting (Wang et al., 2018; Annibaldis et al., 2020), and the obligate role of the RQC pathway in handling stalled ribosomes, further studies are needed to examine the role of the RQC factors in the frame-shifting events in cancer cells.

### Deregulation of ribosome-associated quality control factors in cancer

Despite the lack of mechanistic studies of the RQC factors in cancer biology, the expression of many RQC factors has been shown to be deregulated. Paradoxically, functional studies have implicated both oncogenic and tumor suppressive functions of distinct RQC factors. Sometimes, the same factor, or factors seemingly working at the same steps of RQC, can exert oncogenic and tumor suppressive functions, suggesting complex and context-dependent effects of RQC in cancer cells. For example, ASCC3 (Dango et al., 2011), ABCE1 (Gao et al., 2020), ANKZF1 (Zhou et al., 2019), VCP (Costantini et al., 2021) have been shown to be overexpressed in cancer cells. Functional studies have shown that inhibition of ABCE1 (Gao et al., 2020), ASCC3 (Dango et al., 2011), and VCP (Costantini et al., 2021) may inhibit cancer cell growth and survival, whereas inhibition of NEMF/Clbn (Bi et al., 2005) and ZNF598 (Yang and Gupta, 2018) may have opposite effects. In the case of Pelo, its knockdown can either promote (Pedersen et al., 2014) or inhibit (Gao et al., 2022) cancer cell growth depending on the context. Thus, the involvement of the RQC pathway in cancer is intriguingly complex. The molecular basis of the distinct effects of the RQC factors in cancer biology will be an interesting area for future investigation.

Upon oncogenic transformation, overall translation activity is elevated in part through mTORC1-mediated phosphorylation of 4EBP, S6K, and eEF2K that stimulate translation initiation and elongation. Increased translation may lead to ribosome stalling and collision of certain mRNA containing elongation pausing motifs or when certain aminoacyl-tRNAs are in short supply. *Cancer* cells have evolved RQC modulating strategies, such as resolution of elongation stalling of Myc by eIF5A or programmed frame-shifting to handle amino acid shortage. *Cancer* cells can also regulate the expression of an antiangiogenic form of VEGF-A to promote angiogenesis.

## Translational regulation and ribosome-associated quality control in viral infection

**FIGURE 3:**
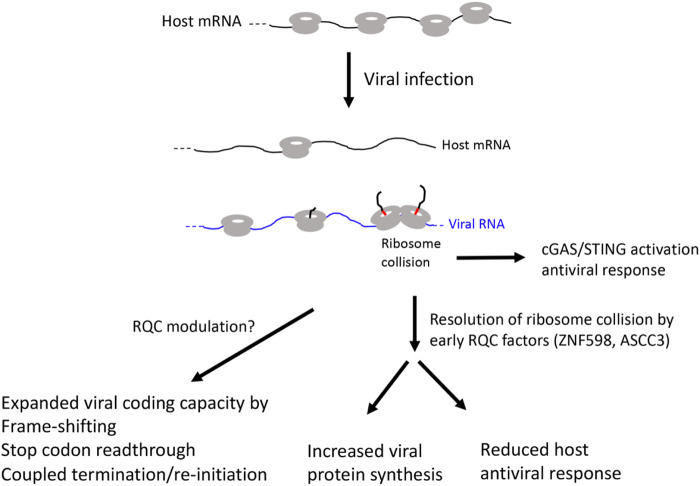
Diagram of possible RQC involvement in viral infection.

A common feature of viruses is their dependency on host translation machinery for completing their life cycle (Jan et al., 2016). Thus, commandeering host ribosomes is critical for viral replication and evolution. To achieve this goal, virus have evolved diverse strategies to subvert host pathways that control translation at the initiation, elongation, and termination steps. Moreover, genome size constrains have driven the evolution of viral strategies to expand coding capacity through leaky scanning, programmed frame-shifting, and stop codon readthrough (Figure 3).

Viral manipulation of translation has been studied extensively at the initiation step. Uncovered strategies include manipulation of mRNA 5’ capping, eIF4F complex assembly, inhibition of 4EBP by mTORC1, etc. (Jan et al., 2016). Various mechanisms of viral manipulation of elongation have also been uncovered, including the deployment of viral mRNA with rare codons (Colson et al., 2013). Such RNAs can deplete certain tRNAs and lead to suppression of host translation. Translation of viral mRNAs with rare codons can also cause ribosome stalling, a prerequisite for frame-shifting by translating ribosomes and synthesis of new viral protein as part of the viral strategies for expanding the coding capacity of the viral genome, which also include leaky scanning, stop codon readthrough, and coupled termination/re-initiation (Jan et al., 2016). Frame-shifting happens when stalled ribosomes slip in -1 or +1 direction as the result of encountering rare codons or RNA structures. In SARS coronovirus, RNA dimerization via kissing loop-loop interactions between pseudoknots has been shown to induce frame-shifting (Imperatore et al., 2022). Viral strategies for manipulation of translation termination have also been revealed. For example, viruses encode eRF-related termination factors, eRF1-binding peptides, or use other viral proteins in a non-canonical manner to bind to eRF1 and regulate the final step in translation (Houck-Loomis et al., 2011).

### Role of ribosome-associated quality control factors in viral infection

Many of the RQC factors have been implicated in viral replication, assembly, and host response. However, it remains uncertain whether the RQC pathways are deployed the same way in viral infected cells as they are used to handle stalled ribosomes in non-infected cells. Perhaps the best evidence supporting RQC pathway involvement in viral infection came from studies of vaccinia virus protein synthesis during infection, where it was shown that viral infection enhanced ubiquitination of Rps20, an event catalyzed by ZNF598 during RQC of collided ribosomes, suggesting that RQC was activated during vaccinia virus infection (Sundaramoorthy et al., 2021). Supporting a functional role of RQC in vaccinia viral infection, viral replication was impaired in cells deficient in ZNF598 activity or expressing a ubiquitination-deficient version of Rps20. Moreover, cellular RQC activity was found declined with vaccinia virus infection, suggesting that co-option of ZNF598 by vaccinia virus confers translational reprogramming that is needed for optimal viral replication (Sundaramoorthy et al., 2021). This result is consistent with an earlier study implicating ZNF598 E3 ligase activity and the ubiquitination of Rps20, but not Rps10, in vaccinia virus replication and the synthesis of vaccinia viral proteins whose encoding mRNAs contain unusual 5’ poly(A) leaders (DiGiuseppe et al., 2018). Interestingly, a recent study showed that mild heat treatment destabilized the RNA-dependent RNA polymerase (also known as Nsp12 protein) encoded by SARS-COV2 virus in a mechanism mediated by ZNF598-dependent ubiquitination of Nsp12 (Maimaitiyiming et al., 2022), suggesting a novel mechanism of action of ZNF598 in cellular response to viral infection and a new way to interfere with SARS-COV2 replication and combat the ongoing COVID-19 epidemic.

The type I interferon (IFN) pathway restricts infection of diverse families of viruses by inducing hundreds of IFN-stimulated genes (ISGs), many of which have direct antiviral activities. In a genetic screen for ISGs that modulate West Nile virus (WNV) infection in interferon β-treated human cells, ASCC3 was identified as a factor that negatively regulate host defense response (Li et al., 2013). Silencing of ASCC3 resulted in upregulation of multiple antiviral ISGs and attenuation of viral infection, whereas ectopic expression of ASCC3 resulted in downregulation of ISGs and increased viral infection. ASCC3 apparently modulated ISG expression in an IRF-3 and IRF-7 dependent manner (Li et al., 2013). A previous study showed that ZNF598 and Rps10 negatively regulate ISG expression and antiviral response (DiGiuseppe et al., 2018). It remains to be seen whether ZNF598/Rps10 and ASCC3 act through similar mechanisms to regulate host antiviral response. A recent study described a surprising role of collided ribosomes in activating the cyclic GMP-AMP synthase-stimulator of interferon genes (cGAS-STING) pathway, which normally senses cytosolic DNA and upregulates ISG expression to activate innate immune system. It was shown that cGAS preferentially binds collided ribosomes *in vitro* and that perturbations that lead to elevation of collided ribosomes and activation of RQC cause redistribution of cGAS from the nucleus to the cytosol *in vivo*, suggesting that translational stress can lead to cGAS activation and antiviral response (Wan et al., 2021). Since ZNF598 and ASCC3 are both early RQC factors that sense ribosome collisions, future studies will test whether handling of ribosome collision is a common mechanism by which ZNF598 and ASCC3 negatively regulate ISG expression and antiviral response in various viral infection conditions.

Other RQC factors have also been implicated in various aspects of viral life cycle. For example, ABCE1 has been implicated in viral mRNA translation and viroid assembly (Anderson et al., 2019). Pelo was first shown to be particularly important for capsid protein expression of *Drosophila* C virus and other viruses in *Drosophila* (Wu et al., 2014). Mechanistically, this is in part due to the role of Pelo in the disassembly of stalled 80S ribosome and clearance of defective viral RNA and proteins (Wu et al., 2014). Consistently, mammalian Pelo has also been shown to be important for DENV and BVDV viral replication (Asad et al., 2018). VCP, another key component of the RQC pathway, has also been implicated in various steps in the viral life cycle, from entry and uncoating to viral egress (Das and Dudley, 2021). However, whether these factors act through canonical RQC activity to modulate viral function remains to be established.

In virus infected cells, virus use multiple strategies to shut off host translation and hijack host translation machineries to promote viral replication and spread. Increased translation of viral mRNA will inevitably lead to ribosome collision. As collided ribosomes can activate the cGAS/STING pathway and induce host antiviral interferon response, viruses use host early RQC factors to resolve ribosome collisions. This will lead to increased viral protein synthesis and reduced host antiviral response. Viruses also use translation elongation related strategies such as stop codon readthrough, frame-shifting, and coupled termination/reinitiation to expand the coding capacity of the compact viral genome. These may also involve RQC manipulation, although the detailed mechanisms remain to be investigated.

## Epilogue

Maintaining an intact yet dynamic proteome requires intricate translational control mechanisms at the initiation, elongation, and termination steps. Recent studies strongly implicate the critical role of RQC mechanisms that handle stalled ribosomes and ribosome collisions in the maintenance of proteostasis, and defects in these mechanisms can contribute to the pathogenesis of major diseases, from neurodegenerative diseases and cancer to viral infection. These studies have begun to offer new RQC-based strategies for the treatment of these major diseases. For example, partial inhibition of the early RQC factors ZNF598 or ASCC3 can inhibit viral protein synthesis and boost host antiviral response. In the case of ZNF598, its partial knockdown has been shown to be beneficial in animal models of AD (Rimal et al., 2021). Our understanding of the mechanisms and function of RQC in mammalian systems remains fragmentary and far from complete. This is mainly because most of the initial studies of RQC were done in yeast and bacteria using artificial mRNA substrates, and there is emerging evidence that mammalian RQC may deploy mechanisms distinct from that in yeast or bacteria. Further studies of RQC in mammalian systems will help address many unanswered questions, including but not limited to the following: 1) Do RQC factors work in similar mechanisms and pathways in cancer cells or during viral infection, as during RQC in normal cells? 2) What are the upstream signaling mechanisms that regulate RQC in settings of neurodegeneration, oncogenesis, and infection? 3) What are the key targets of the RQC pathway in the above disease settings? 4) How could the RQC factors that work in seemingly similar RQC processes play distinct role in cancer biology? 5) Can the viral factors deployed by viruses to manipulate host translational machinery be leveraged to treat cancer or neurodegenerative diseases? Answers to these questions may lead to new understanding of translational control mechanisms in proteostasis regulation under normal conditions and offer new clues on how this fresh insight can be harnessed to combat major diseases.
